# Dual effect of a single nucleotide polymorphism in the first intron of the porcine *Secreted phosphoprotein 1 *gene: allele-specific binding of C/EBP beta and activation of aberrant splicing

**DOI:** 10.1186/1471-2199-10-96

**Published:** 2009-10-21

**Authors:** Eduard Muráni, Siriluck Ponsuksili, Hans-Martin Seyfert, Xuanming Shi, Klaus Wimmers

**Affiliations:** 1 Research Institute for the Biology of Farm Animals (FBN), Research Unit Molecular Biology, Wilhelm-Stahl-Allee 2, 18196 Dummerstorf, Germany; 2 Research Institute for the Biology of Farm Animals (FBN), Research Group Functional Genomics, 18196 Dummerstorf, Germany

## Abstract

**Background:**

Secreted phosphoprotein 1 (SPP1 or Osteopontin, OPN) is a multifunctional matricellular glycoprotein involved in development and regeneration of skeletal muscle. Previously, we have demonstrated that porcine *SPP1 *shows breed-related differential mRNA expression during myogenesis. With the aim to identify putative contributing *cis*-regulatory DNA variation we resequenced the 5' upstream region of the gene in the respective breeds Pietrain and Duroc. We found two single nucleotide polymorphisms (SNP; [GenBank:M84121]: g.1804C>T and g.3836A>G). We focused our investigation on the SNP g.3836A>G, because *in silico *analysis and knowledge about the regulation of *SPP1 *suggested an effect of this SNP on a CCAAT/enhancer binding protein beta (C/EBPβ) responsive transcriptional enhancer.

**Results:**

Using electrophoretic mobility shift assay we demonstrated that, similar to human *SPP1*, the 3' terminal end of the first intron of porcine *SPP1 *harbors a C/EBPβ binding site and showed that this binding site is negatively affected by the mutant G allele. Genotyping of 48 fetuses per breed revealed that the G allele segregated exclusively in Duroc fetuses with a frequency of 57 percent. Using real-time quantitative PCR we showed that, consistent with its negative effect on a transcriptional enhancer element, the G allele tends to decrease mRNA abundance of *SPP1 *in the fetal *musculus longissimus dorsi *(~1.3 fold; P ≥ 0.1).

Moreover, we showed that the SNP g.3836A>G leads to ubiquitous aberrant splicing of the first intron by generating a *de novo *and activating a cryptic splice acceptor site. Aberrantly spliced transcripts comprise about half of the *SPP1 *messages expressed by the G allele. Both aberrant splice variants differ from the native transcript by insertions in the leader sequences which do not change the reading frame of *SPP1*.

**Conclusion:**

At the 3' terminal end of the first intron of the porcine *SPP1 *we identified a unique, dually functional SNP g.3836A>G. This SNP affects the function of the *SPP1 *gene at the DNA level by affecting a C/EBPβ binding site and at the RNA level by activating aberrant splicing of the first intron, and thus represents an interesting DNA-marker to study phenotypic effects of *SPP1 *DNA-variation.

## Background

Secreted phosphoprotein 1 (SPP1 or Osteopontin, OPN) is a matricellular glycoprotein mediating cell-adhesion, -migration, -survival and -signalling via integrin and CD44 receptors [1]. In line with its versatility and widespread expression SPP1 has been linked with various physiological and pathological events, amongst others development and regeneration of skeletal muscle [2,3]. *SPP1 *is regulated by the muscle regulatory factors MYOD and MYF5 [4] and has been shown to be expressed *in vitro *in myoblasts and myotubes [3,5]. Uaesoontrachoon *et al*. [3] demonstrated that soluble SPP1 stimulates proliferation of myoblasts whereas SPP1 deposited in extracellular matrix promotes their differentiation. Recently we showed upregulation of *SPP1 *mRNA expression in prenatal *musculus longissimus dorsi *(M.l.d.) at 35 and 63-77 days *post conception *(dpc) in pigs, i.e. at the time points of the first and second myogenic wave respectively, thus providing additional, *in vivo*, evidence supporting involvement of SPP1 in myogenesis. In addition we showed consistent elevation of *SPP1 *mRNA level during myogenesis in the pig breed Duroc compared to breed Pietrain, the latter showing higher muscularity and higher proportion of fast twitch glycolytic fibers postnatally [6]. Moreover we mapped quantitative trait loci for the proportion of fast twitch glycolytic fibers and pH of M.l.d. 45 minutes *post mortem *on porcine chromosome 8 close to *SPP1 *position [7]. To detect polymorphisms that contribute to the breed-related differential mRNA expression of *SPP1 *and which are associated with differential microstructural and biophysical muscle properties we performed SNP screening of the upstream regulatory region. We identified and functionally characterized a SNP located in the 3' terminal end of the first intron in an evolutionarily conserved transcriptional enhancer [8,9].

## Results

### Identification and *in silico *characterization of SNP g.3836A>G

**Figure 1:**
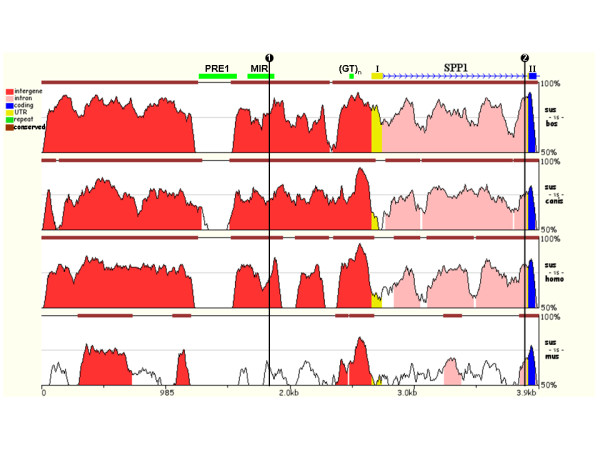
**Evolutionary conservation and variation of the sequence of the upstream region of porcine *SPP1***. The position of exons labelled by numbers in roman numerals, and of repetitive elements labelled by their identity (Porcine repetitive element 1, PRE1; Mammalian interspersed repetitive element, MIR; GT-microsatellite, (GT)_n_) is indicated at the top. The arrowed line shows direction of the transcription. Beneath stacked-pairwise conservation profiles between porcine and corresponding bovine, canine, human and murine sequences generated using the Mulan tool are shown. Bars above the conservation profiles indicate evolutionarily conserved regions (>70% identity; >100 bp). Numbered lines show position of the identified SNP ([GenBank:M84121]: 1. g.1804C>T; 2. g.3836A>G)

We resequenced ~3.8 kb of the 5'-upstream region of the porcine *SPP1 *including 5'-flanking region and promoter, exon1, intron1, as well as exon2 and identified two SNP: a C>T SNP in the 5'-flanking region ([GenBank:M84121]: g.1804C>T) and an A>G SNP in the first intron ([GenBank:M84121]: g.3836A>G). Phylogenetic footprinting of the 5'-upstream region indicated that the g.1804C>T SNP resides in an evolutionarily poorly conserved sequence of a repetitive element (Mammalian Interspersed Repeat; MIR) and thus most likely possesses no function. In contrast the g.3836A>G SNP is located in an evolutionarily conserved region at the 3' terminal end of the first intron (Figure 1) that has been shown to harbor a strong transcriptional enhancer in the pig and in human [9,8]. The SNPInspector software predicted the elimination of binding sites of four transcription factors by this SNP including CCAAT/enhancer binding protein (C/EBP), adenovirus E4 promoter binding protein (E4BP4), thyrotrophic embryonic factor (TEF) and fork head homologous X (FHXB). Interestingly, a CCAAT/enhancer binding protein beta (C/EBPβ) binding site, overlapping the site corresponding to the g.3836A>G SNP site in the human *SPP1*, has been shown to be important for the activity of the intronic enhancer [8].

### The C/EBPβ binding site at the 3' terminal end of the first intron of *SPP1 *is evolutionarily conserved in the pig and is negatively affected by the SNP g.3836A>G

**Figure 2:**
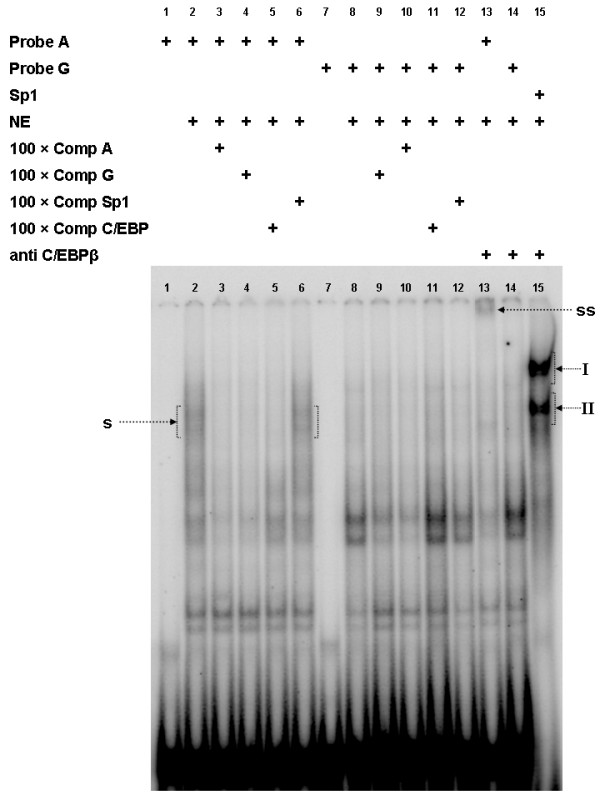
**EMSA and supershift assay using probes allelic for the g.3836A>G SNP and nuclear extracts from *longissimus dorsi *muscle of 91 day old fetuses**. Addition of nuclear extracts (NE), labelled probes, 100-fold molar excess of different unlabelled competitors and antibody is indicated above each lane (Comp A: competitor bearing the wild type A allele; Comp G: competitor bearing the mutant G allele; Comp Sp1: probe bearing the consensus Sp1 binding motif as unspecific competitor; Comp C/EBP: probe bearing the consensus C/EBP binding motif). s indicates specific DNA:protein complex involving C/EBPβ; ss indicates supershifted complex; roman numerals (I-II) indicate DNA:protein complex formed by labelled probe bearing the consensus Sp1 binding motif

In order to examine the effect of the SNP g.3836A>G on the binding of nuclear proteins, specifically of C/EBPβ, we performed competitive electrophoretic mobility shift assay (EMSA) and supershift assay respectively. Allelic probes centered around the g.3836A>G SNP showed distinct differences in their ability to bind nuclear proteins from porcine fetal skeletal muscle (Figure 2). The labelled probe containing the wild-type allele A formed a DNA:protein complex (designated "s"; Figure 2, lane 2) which was completely absent in all binding reactions containing the labelled mutant probe G (Figure 2, lanes 8-12). Complex "s" was specifically competed by the addition of excess of the corresponding unlabelled probe A (Figure 2, lane 3) or unlabelled probe bearing consensus C/EBP binding motif (Figure 2, lane 5) respectively, but was unaffected by the addition of a nonspecific competitor probe, in this case bearing consensus Sp1 binding motif (Figure 2, lane 6). Addition of C/EBPβ antibody to a binding reaction containing the labelled probe A caused disappearance of the complex "s" and caused an evident supershifted complex (designated "ss"; Figure 2, lane 13). Addition of the antibody to a binding reaction containing the labelled probe bearing the Sp1 binding motif had no effect on the DNA:protein complexes (designated "I" and "II"; Figure 2, lane 15) and also produced no supershifted complex in the binding reaction containing the labelled mutant probe G (Figure 2, lane 14), demonstrating specificity of the supershifted complex "ss". Our EMSA experiment thus provides clear evidence that C/EBPβ participated on the formation of the complex "s" and consequently provides evidence that the C/EBPβ binding site present in human *SPP1 *is evolutionarily conserved in porcine *SPP1*. The heterogeneity of the complex "s" is most likely due to the presence of multiple C/EBPβ isoforms or heterodimers with other members of the C/EBP family.

**Figure 3:**
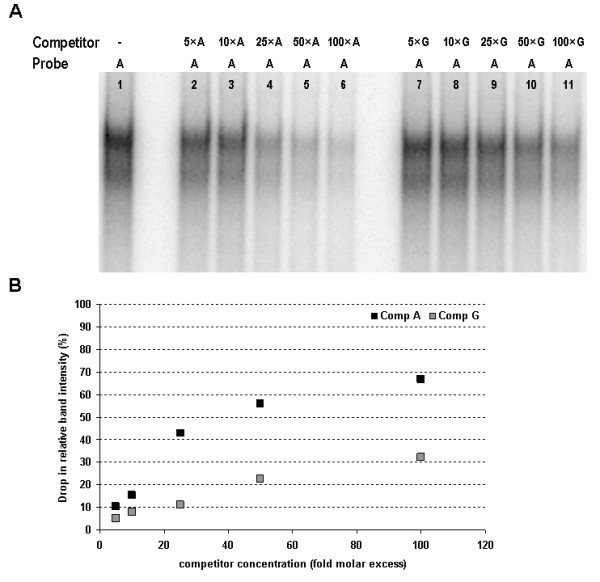
**Analysis of binding affinity of the probes allelic for the SNP g.3836A>G by competitive EMSA using nuclear extract from cells overexpressing N-terminally truncated bovine C/EBPβ**. (A) Competitive EMSA was performed using labelled probe A and 5-100 fold molar excess of cold competitor probes A (lanes 2-6) and G (lanes 7-11) respectively. In the first lane EMSA was performed without competitor. (B) The relative band intensity (EMSA without competitor representing the basal level of 100%) plotted vs. increasing concentrations of allelic competitor probes.

Competitive inhibition of the complex "s" by addition of 100-fold molar excess of the unlabelled mutant probe G indicates that binding of C/EBPβ is not completely abolished by the SNP g.3836A>G (Figure 2, lane 4). To characterize the effect of the g.3836A>G SNP on the C/EBPβ binding site more precisely we performed competitive EMSA using nuclear extracts from cells overexpressing N-terminally truncated bovine C/EBPβ (ΔN-C/EBPβ; Figure 3A). The ΔN-C/EBPβ spans the bovine C/EBPβ from amino acids 155 to 348, i.e. it retains the DNA-binding domain and the bZIP domain [10]. The complex formed between the labelled probe A and ΔN-C/EBPβ was competed away using 5-100 fold molar excess of unlabelled probe A and G respectively. The decrease in relative intensity of the complex as a consequence of increasing unlabelled competitor concentration is an indication of the efficiency of displacement, thereby reflecting the relative affinity of the respective alleles to C/EBPβ. The efficiency of displacement of the cold competitor probe G is more than twofold lower compared to the cold competitor probe A demonstrating that the mutation reduced the affinity of the binding site to C/EBPβ in excess of twofold (Figure 3B).

### SNP g.3836A>G decreases mRNA abundance of *SPP1 *in fetal M.l.d

**Figure 4:**
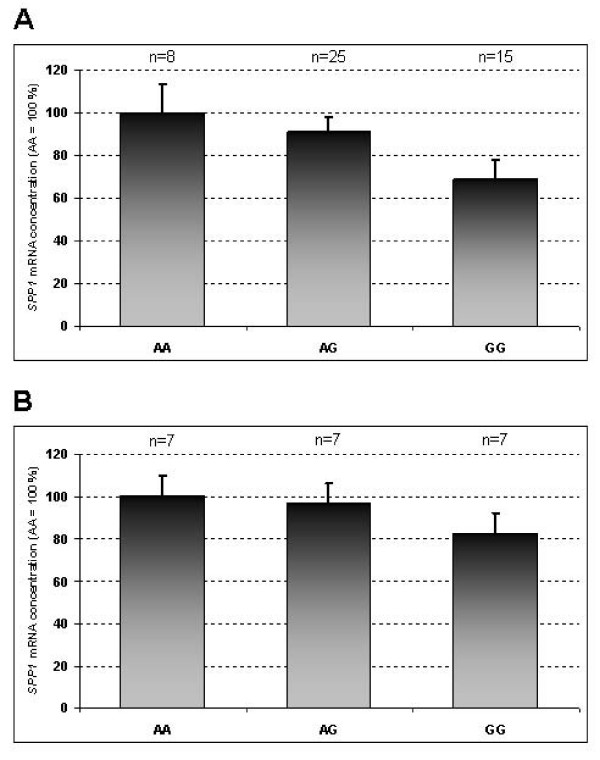
**mRNA expression of porcine *SPP1 *in fetal longissimus dorsi muscle according to the genotype at the SNP g.3836A>G**. (A) Results of the across-stage analysis of pooled data of each 12 Duroc fetuses per stage 49, 63, 77 and 91 days *post conception *(dpc). (B) Results of an independent analysis of 7 Duroc fetuses (91 dpc) per genotype class. Each bar shows the least square means ± standard error for a genotype class. The number of individuals per genotype class is indicated above the corresponding bar.

To examine whether the reduced affinity of the C/EBPβ binding site due to SNP g.3836A>G leads to differential mRNA expression of *SPP1 in vivo *we genotyped 12 fetuses per each breed (Pietrain and Duroc) and stage (49, 63, 77 and 91 dpc) with expression data from a previous study [6]. The SNP turned out to be fixed for the ancestral A allele in the Pietrain fetuses, segregating only amongst the Duroc fetuses with a frequency of 57% of the mutant G allele. The analysis of the effect of the SNP on *SPP1 *mRNA expression was therefore performed within the Duroc breed. The data were pooled across the four stages, which was accounted for in the statistical model. The results revealed a decreasing trend in the *SPP1 *mRNA abundance associated with the mutant G allele (P = 0.08; Figure 4A). Quantification and separate analysis of *SPP1 *mRNA expression in M.l.d. of additional seven 91 dpc fetuses per each genotype class revealed similar, although less pronounced, decreasing tendency (Figure 4B). The decreasing trend in mRNA abundance of *SPP1 *associated with the G allele is consistent with the evidence of an altered function of the mutated C/EBPβ binding site which normally acts as a transcriptional enhancer. With regard to the breed-related differences in the mRNA expression of *SPP1 *gene during myogenesis the upregulation in Duroc is apparently caused by factors different from SNP g.3836A>G.

### SNP g.3836A>G leads to ubiquitous aberrant splicing of porcine *SPP1 *in the 5' untranslated region

**Figure 5:**
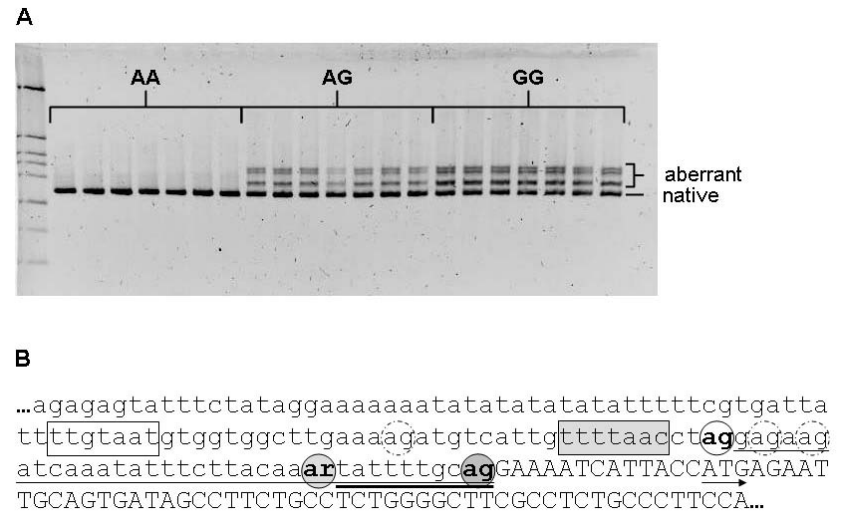
**Identification of aberrant splicing of the porcine *SPP1 *mRNA activated by the g.3836A>G SNP**. (A) High-resolution agarose electrophoretic analysis of RT-PCR-amplified fragment of porcine *SPP1 *spanning exons 1 to 6 using seven *longissimus dorsi *muscle cDNA from 91 day old fetuses per each genotype class. (B) Nucleotide sequence of the 3' terminal end of the first intron and exon 2 of porcine *SPP1 *[GenBank:M84121]. Native and the identified *de novo *and cryptic splice acceptor sites are shown in bold letters in darkly shaded, lightly shaded and open circles respectively. The SNP position is indicated in IUPAC ambiguity code. Sequences exonized by the *de novo *and cryptic splice acceptor sites are underlined by thick and thin line respectively. Additional potential splice acceptor sites are indicated by dashed line-circles. Putative authentic and cryptic branch sites are indicated by shaded and open boxes respectively. The intron and exon sequences are shown in lower and upper case letters respectively. The translation start is indicated by an arrow.

**Figure 6:**
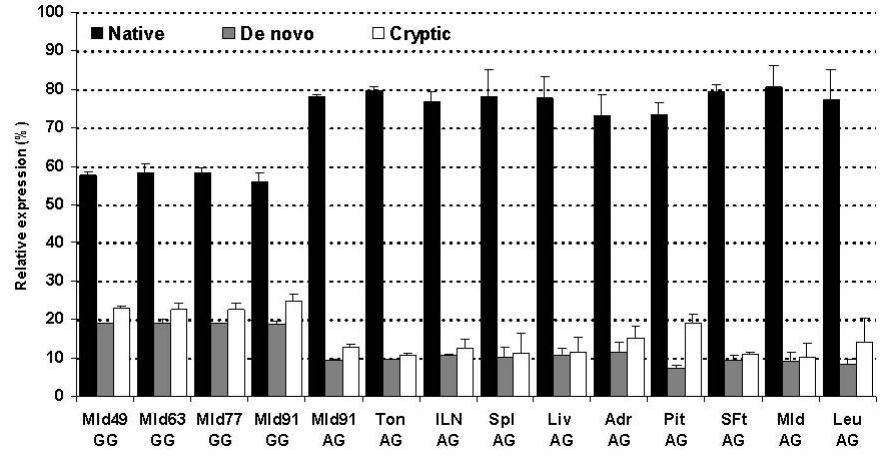
**Relative expression of native and aberrant splice variants of the porcine *SPP1 *mRNA across various tissues**. Each bar shows the mean ± standard deviation per splice variant. Source tissue and genotype is indicated below. Mld 49, 63, 77 and 91: fetal *longissimus dorsi *muscle at 49, 63, 77 (each n = 2) and 91 (each genotype n = 7) days *post conception*; Ton: tonsil; ILN: intestinal lymph node; Spl: spleen; Liv: Liver; Adr: adrenal; Pit: pituitary; SFt: subcutaneous fat; Mld: adult *longissimus dorsi *muscle; Leu: leukocyte; (each adult tissue n = 3)

The mutant G allele generates a potential splice acceptor site (3'ss) only 10 bp upstream of the naturally occurring intron1/exon2 junction. To prove whether this potential *de novo *3'ss is active *in vivo *we performed RT-PCR using the M.l.d. cDNA samples of 91 day old fetuses described in the preceding paragraph. As shown in Figure 5A carriers of the wild-type A allele expressed only one splice variant whereas multiple splice variants were detected in samples carrying the G allele. Cloning and sequencing revealed that the mutant G allele causes aberrant splicing of the first intron thus generating heterogeneity in the 5' untranslated region. Two aberrantly spliced transcript variants were identified: one variant with 10 bp longer leader sequence spliced at the *de novo *3'ss generated by the G allele, and another with a 36 bp longer leader sequence spliced at a cryptic 3'ss located in the first intron 24 bp upstream of the g.3836 SNP site (Figure 5B). The insertions do not lead to a change in the *SPP1 *reading frame and also do not generate open reading frames upstream of the translation start site that could reduce translational efficiency. Inspection of the 3' terminal region of the first intron revealed two additional potential 3'ss between the authentic 3'ss and a putative branch site and one additional potential 3'ss between the cryptic 3'ss and a putative cryptic branch site. To describe the splicing pattern in more detail we quantified relative amounts of the splice variants in M.l.d. across different fetal stages and across various tissues/cell types of adult animals including tonsil, intestinal lymph node, liver, spleen, pituitary, adrenal gland, M.l.d., subcutaneous fat and leukocyte by fragment analysis on MegaBACE capillary sequencer. Only heterozygous adult carriers were available. We found aberrant splicing of *SPP1 *mRNA across all examined tissues of the carriers of the mutant G allele. No additional splice variants were detected by the more sensitive analysis on the capillary sequencer. The relative amounts of the three splice variants were allele dosage dependent; in homozygous GG animals the proportion of native: *de novo*: cryptic splice variants was about 56: 19: 25 and in heterozygous AG animals the proportion was about 78: 9: 13 (Figure 6). We employed the MaxEntScore tool, implementing maximum entropy modelling (ME), to examine the strength of the different identified active and potential 3'ss *in silico*. Vorechovsky [11] showed that this method performs best in the prediction of the utilization of aberrant 3' ss. MaxEntScore revealed that the mutation weakened the 3'ss, with the ME score of the authentic 3'ss dropping from 7.86 to 3.13 for the mutated authentic 3'ss respectively. The ME score of the cryptic 3'ss (6.9) is higher than that of the *de novo *3'ss (1.33). The three potential 3'ss had low scores (in direction from 3' to 5': -8.81, -10.09 and 0.48) in accord with the fact that they are not used in *vivo*.

## Discussion

The total number of myofibers and to some extent their metabolic and contractile properties are determined during the process of myogenesis. Insights into the regulation of this process will consequently bring about better knowledge of factors affecting postnatal growth and function of skeletal muscle. We previously discovered breed-related differences in mRNA expression of porcine *SPP1 *during myogenesis [6] and hypothesized that this variation might be caused by *cis*-regulatory DNA-variation. *Cis*-regulatory DNA variation has been shown to affect a large proportion (~20%) of genes [12] and in fact has already been described in human *SPP1 *[13,14]. We identified an A>G SNP at position g.3836 at the 3' terminal end of the first intron of porcine *SPP1*. *In silico *analysis of the polymorphic sequence and knowledge about the regulation of *SPP1 *in pigs and human suggested an effect of the SNP on a C/EBPβ responsive transcriptional enhancer. Using competitive EMSA and supershift assay we showed that the C/EBPβ binding site found in human is present also in the pig and is negatively affected by the mutation. As a consequence of the reduced affinity of the C/EBPβ binding site the activity of the transcriptional enhancer is reduced, at least in fetal M.l.d., as indicated by the downregulation of *SPP1 *mRNA expression by fetuses carrying the G allele. Conservation of the C/EBPβ binding site at the 3' terminal end of the first intron of human and porcine *SPP1 *points to an important function of this element in the regulation of *SPP1*. The relevance of C/EBPβ in positive regulation of mRNA expression of *SPP1 *is further emphasized by the identification of additional functional C/EBP transcriptional enhancer elements in the promoter of murine and human *SPP1 *[15,16]. C/EBPs are a family of transcription factors involved in the regulation of proliferation and differentiation of diverse cell types, and play pivotal roles in a number of processes including adipogenesis [17]. However, an involvement or a precise function of C/EBPs in myogenesis has not been described so far.

We demonstrate that the SNP g.3836A>G is functional also on the RNA level and causes aberrant splicing of the first intron. We found that this effect is ubiquitous with regard to temporo-spatial distribution which is in accord with the fact that it creates a general splicing signal. Naturally occurring DNA-variation affecting splicing represents a valuable resource to identify sequence signals involved in the regulation of this process. The g.3836A>G SNP exhibits several features of *de novo *3'ss activating aberrant splicing identified by Kralovicova *et al*. [18] and Vorechovsky [11]. It creates an AG dinucleotide in intron in polypyrimidine tract of the authentic 3'ss by introducing a guanine residue, has an uridine in position +1 relative to the *de novo *3'ss and activates a cryptic 3'ss. Vorechovsky [11] has shown that authentic counterparts of *de novo *3'ss are intrinsically weak. In the case of the affected 3'ss of *SPP1 *this is likely a consequence of the two concurrent functional constraints on the sequence of the 3' terminal end of the first intron; on one hand to provide a C/EBPβ responsive transcriptional enhancer and on the other to provide the canonical splicing acceptor signals. The *in silico *predicted strength of the mutated authentic 3'ss compared to the cryptic 3'ss and their utilization *in vivo *are reversed. An explanation for the lower utilization of the cryptic 3'ss compared to the mutated authentic 3'ss *in vivo *may be a suboptimal sequence of the putative cryptic branch site, in contrast to the predicted authentic branch site which perfectly matches with the consensus sequence YNYURAY (Figure 5B). Another explanation might be that the cryptic 3'ss is silenced by a splicing silencer or *vice versa *that the authentic 3'ss might be augmented by a splicing enhancer. Kralovicova and Vorechovsky [19] showed that *in vivo *selection of aberrant splice sites is extensively controlled by auxiliary splicing signals.

Mutations that affect hnRNA splicing account for up to 50% of disease-causing gene alterations in human and potentially represent the most frequent cause of hereditary disorders. Some 218 unique aberrant 3'ss, activated by disease-causing mutations in 131 genes, are presently known in human [11]. Our results show that about half of the *SPP1 *messages expressed by the G allele comprise aberrantly spliced transcripts. Considering the versatile function of SPP1 and the ubiquitous expression of the aberrant splice variants it could be speculated that the SNP g.3836A>G might have pleiotropic effects on various traits including growth, reproduction and immune defence. However the relatively high frequency of the G allele argues against a major negative phenotypic effect, because in that case the G allele would be quickly eliminated given the high selection pressure on commercial pigs. Furthermore, the aberrant splicing induced by the SNP g.3836A>G does not change primary structure of the SPP1 protein. In fact, the aberrant splicing may counteract the negative effect of the G allele on mRNA expression of *SPP1 *by enhancing translational efficiency or RNA stability. Aberrantly spliced mRNA isoforms of the human insulin gene with longer 5'-leader sequence, induced by a SNP in its first intron, were reported to generate more proinsulin *in vitro *compared to the native transcripts [20].

## Conclusion

Functional characterization of the SNP g.3836A>G revealed that it has two effects; it negatively affects a C/EBPβ binding site and activates aberrant splicing of the first intron. However, although the SNP g.3836A>G is functional it most likely does not represent the causative mutation responsible for the previously observed breed related differences in *SPP1 *mRNA expression. Nevertheless, the dual effect of the SNP on *SPP1 *function renders it as an interesting DNA-marker for association studies concerning muscle-related, growth, reproduction, and immune defence traits.

## Methods

### Identification of SNP and splice variants

The target sequence of the porcine *SPP1 *gene [GenBank:M84121] was amplified in six overlapping PCR fragments using standard PCR conditions and each six Pietrain and Duroc DNA samples respectively. A standard PCR reaction mixture contained 100 ng genomic DNA, 0.2 μM of each primer, 50 μM of each dNTP and 0.5 U SupraTherm Taq Polymerase in 1× supplied PCR-buffer containing 1.5 mM MgCl_2 _(Genecraft, Lüdinghausen, Germany). The temperature profile consisted of 40 cycles of denaturation at 95°C for 15 s, annealing at T_a _for 30 s and extension at 72°C for 30 s for each <0.5 kb. Amplification products were subsequently pooled within breed and purified using the NucleoSpin Extract II kit (Macherey-Nagel, Düren, Germany).

To detect alternative splice variants a cDNA fragment spanning exons 1-6 was amplified in a standard PCR reaction and cloned using the pGEM-T vector (Promega, Mannheim, Germany). The PCR products and plasmids were sequenced using Big Dye Terminator Cycle sequencing kit V1.1 (Applied Biosystems, Darmstadt, Germany) and analyzed on ABI 310 or MegaBACE 750 automated sequencer.

The g.3836A>G SNP was amplified in a standard PCR reaction and genotyped using single strand conformation polymorphism (SSCP) visualized by silver staining after electrophoresis performed for 5 hours at 5°C on a 12% native polyacrylamide (49:1 AA: Bis) gel in 0.5 × TBE buffer. Sequences and annealing temperature (T_a_) of primers used for comparative sequencing, genotyping and RT-PCR are given in Additional File 1: Table S1.

### Tissue collection, RNA isolation and cDNA synthesis

Sampling of fetal M.l.d. was described in detail previously [6]. Briefly, immediately after exsanguination of the sows the uteri were recovered and the embryos/fetuses were quickly removed, weighed and the M.l.d. dissected. Adult tissue samples from performance tested animals were collected in our experimental abattoir. After dissection samples were quickly frozen in liquid nitrogen and stored at -80°C for later analysis. Total RNA was isolated using TRI Reagent (Sigma, Taufkirchen, Germany). After DNaseI treatment (Roche, Mannheim, Germany) the RNA was cleaned up using the NucleoSpin RNA II Kit (Macherey-Nagel). First strand cDNA was synthesized using SuperScriptIII MMLV reverse transcriptase (Invitrogen, Karlsruhe, Germany) in a reaction containing 1 μg RNA and 500 ng of oligo (dT)_11_VN primer, according to the manufacturer's protocol.

### Quantification of total transcript level and relative amounts of splice variants

Total transcript level of *SPP1 *and of the reference gene *RPL32 *were quantified by real-time quantitative PCR (qPCR) performed on a LightCycler 1.0 System using the LightCycler FastStart DNA Master SYBR^plus ^Green I (Roche). The amplification was conducted in duplicate according to manufacturer's instructions using 200 μM of each primer. The temperature profiles consisted of an initial denaturation step at 95°C for 10 min and 45 cycles consisting of denaturation at 95°C for 10 s, annealing at 60/55°C for *SPP1*/*RPL32 *and extension/fluorescence acquisition at 72°C for 15 s. For both assays threshold cycles were converted to copy numbers using a standard curve generated by amplifying serial dilutions of an external PCR standard (10^7 ^- 10^1 ^copies). To account for variation in RNA input and efficiency of reverse transcription the calculated *SPP1 *mRNA copy numbers were normalized by dividing with a normalization factor derived from the expression of the reference gene.

To determine relative amount of splice variants the cDNA fragment described above was amplified using a FAM labelled primer R1F (Additional file 1: Table S1), separated on a MegaBACE 750 capillary sequencer and peak heights were measured using the MegaBACE Fragment Profiler v1.2 software (GE Healthcare, Munich, Germany). The relative quantity of a splice variant was calculated by dividing the corresponding peak height by the sum of peak heights corresponding to all three splice variants.

### Electrophoretic mobility shift assay

Nuclear proteins from M.l.d. of 91 old fetuses were prepared essentially as described by Fürbass *et al*. [21]. Cells (murine mammary epithelial cell line HC11) overexpressing N-terminally truncated bovine C/EBPβ (ΔN-C/EBPβ) and nuclear extracts from these were prepared as described by Yang *et al*. [22]. Double-stranded probes were prepared by annealing a sense oligo with a shorter antisense oligo (Additional file 1: Table S2) serving as a primer of a Klenow fill-in reaction containing 5U enzyme (Fermentas, St. Leon-Rot, Germany), 1× buffer supplied by the manufacturer and 100 μM each dNTP or 20 μCi [α-^32^P]dATP for labelling. Nuclear extracts (~3 μg) were incubated with 80 fmol labelled probes (40 fmol labelled probes for EMSA using ΔN-C/EBPβ) in a binding mixture containing 10 mM HEPES-KOH pH 7.9, 50 mM KCl, 0.1 mM EDTA, 0.5 mM DTT, 1 μ poly(dI-dC), 10% glycerol and 1× protease inhibitor cocktail (Roche) at 20°C for 20 min. For competition experiments unlabelled probes were added 10 min prior to addition of labelled probes. For supershift assay 4 μg of an antibody against C/EBPβ (sc-150X, Santa Cruz) was included into the binding reaction. Samples were subsequently subjected to electrophoresis using native 6% polyacrylamide (30:1 AA: Bis) gels in 0.5 × TBE buffer at 20°C. After electrophoresis gels were dried on Whatmann paper, exposed overnight to phosphor storage screens and analysed on a STORM 840 PhosphorImager (Molecular Dynamics, Krefeld, Germany). Band intensities (peak heights) were measured using the ImageQuant TL v2005 software (GE Healthcare). The difference in the binding affinity between the allelic probes was estimated as the average of the ratio of the intensity drop caused by addition of wild type A competitor divided by the intensity drop caused by mutant competitor G for each competitor concentration.

### Data analysis

Phylogenetic footprinting was performed using the Mulan online tool accessible at the NCBI DCODE.org Comparative Genomics Developments website . The corresponding bovine, canine, human and murine sequences were retrieved from USCS genome browser . Simple and interspersed repeats were identified using Repeatmasker . Transcription factor binding sites and the effect of the g.3836A>G SNP on these were predicted by the SNPInspector software .

The strength of the potential splice donor sites *in silico *was examined using MaxEntScan . To detect open reading frames of the sequences of the different splice variants ORF finder was employed .

The effect of g.3836A>G genotype on mRNA expression of *SPP1 *was analyzed using general linear model (PROC GLM; SAS V9.1, SAS Inst. Inc., Cary, NC) including fixed effects of genotype, stage and their interaction for across stage analysis and fixed effect of genotype for the separate analysis within stage 91 dpc. Least square mean values of the genotypes were compared by a t-test, and the P-values were adjusted by a Tukey-Kramer correction.

## Authors' contributions

EM carried out EMSA, data analysis, and drafted the manuscript. SP provided input to design of the study, participated in sampling and assisted drafting the manuscript and revising it critically for scientific content. HMS and XMS generated the cells expressing ΔN-C/EBPβ, prepared the nuclear extracts, and provided intellectual input for giving final shape of the manuscript. KW significantly contributed to the concept, design and coordination of the study and in drafting the manuscript. All authors read and approved the final manuscript.

## Supplementary Material

Additional file 1 **Table S1 and Table S2**. Primer and probe sequence information Click here for file
