# Supplementary material for: Dual effect of a single nucleotide polymorphism in the first intron of the porcine Secreted phosphoprotein 1 gene: allele-specific binding of C/EBP beta and activation of aberrant splicing
Source: BMC Mol Biol. 2009 Oct 21;10:96. doi: 10.1186/1471-2199-10-96 (PMC2773772; doi:10.1186/1471-2199-10-96)
Supplement: Additional file 1 — Table S1 and Table S2. Primer and probe sequence information [file 1471-2199-10-96-S1.DOC]

**Table S1. Oligonucleotide sequences**

| Experiment | Oligo | Ta (Cº) | Sequence (5´-3´) |
| --- | --- | --- | --- |
| SNP | P1F | 57 | cttctcttggcctccgtgtt |
| screening | P1R |  | tgcacatcaacaggctaattg |
|  | P2F | 55 | tcacaactgagaaagggagatg |
|  | P2R |  | aggctattaaaatgcgaccaga |
|  | P3F | 63 | catctggtcgcattttaatagcc |
|  | P3R |  | gccagcttctctccatttcg |
|  | P4F | 63 | gacatgaacgtggatttgaacg |
|  | P4R |  | gccctggatctgacatgctt |
|  | P5F | 57 | cgctgatggttgctgtcgtag |
|  | P5R |  | atctccagccgtctaaacctaa |
|  | P6F | 65 | aagctgaaaaatcgcccttg |
|  | P6R |  | actcactggaagggcagagg |
|  |  |  |  |
| Genotyping | G1F | 62 | Aatgtggtggcttgaaaagatg |
|  | G1R |  | actcactggaagggcagagg |
|  |  |  |  |
| RT-PCR | R1F | 58 | TGACAGCCGCATCAGCATT |
|  | R1R |  | CGTCGTCCACATCGTCTGTT |
|  |  |  |  |
| qPCR | Spp1F | 60 | TGACAGCCGCATCAGCATT |
|  | Spp1R |  | CGTCGTCCACATCGTCTGTT |
|  | RPL32F | 55 | AGCCCAAGATCGTCAAAAAG |
|  | RPF32R |  | TGTTGCTCCCATAACCAATG |

**Table S2. Sequences of oligonucleotides used to prepare double-strand probes for EMSA**

| Oligo | Sequence (5´-3´) |
| --- | --- |
| Probe A | caaatatttcttacaaaAtattttgcaggaaaatc |
| Probe G | caaatatttcttacaaaGtattttgcaggaaaatc |
| Labelling oligo | GATTTTCCTGCAAAATA |
|  |  |
| Comp Sp1 | ATTCGATCGGGGCGGGGCGAGC |
| Labelling oligo | GCTCGCCCCGCCC |
|  |  |
| Comp C/EBP | TGCAGATTGCGCAATCTGCA |
| Labelling oligo | TGCAGATTGCGCAATC |
